# Pediatric Spigelian Hernia and Spigelian–Cryptorchidism Syndrome: An Integrative Review

**DOI:** 10.3390/children12091120

**Published:** 2025-08-25

**Authors:** Javier Arredondo Montero, María Rico-Jiménez

**Affiliations:** 1 Pediatric Surgery Department, Complejo Asistencial Universitario de León, 24008 León, Castilla y León, Spain; 2 Pediatric Surgery Department, Hospital Universitario Niño Jesús, 28009 Madrid, Spain

**Keywords:** Spigelian hernia, traumatic abdominal wall hernia, cryptorchidism, Spigelian-Cryptorchidism Syndrome, handlebar hernia

## Abstract

Spigelian hernia (SH) is an infrequent aponeurotic defect in Spiegel’s semilunar line. The literature on pediatric SH is scarce. A comprehensive review of the previous literature was conducted. Eligible studies were identified by searching primary medical bibliography databases, and a pooled analysis of published case-level data was performed. Medians and interquartile ranges were used to describe the quantitative variables and proportions for categorical variables. The Kruskal–Wallis, Mann–Whitney U, and Fisher’s exact tests were used to compare group variables. Spearman’s and Pearson’s correlation analyses were used to assess the degree of correlation between variables, while Cramér’s V was applied to evaluate the degree of association among the variables. A *p*-value < 0.05 (two-tailed) was considered statistically significant. Our search identified 82 publications reporting on 123 patients (106 male, 86.2%), with an age range of 0–21 years. Forty-seven patients (38.2%) had a left-sided SH, fifty-six (45.5%) had a right-sided SH, and thirteen (10.6%) had a bilateral SH. Traumatic SH, mostly from bicycle injuries, accounted for 45 cases (36.6%), while 41 (33.3%) were associated with undescended testis (UDT). In this series of published cases, hernia incarceration/strangulation (I/S) was reported in 15 patients (12.2%), who were significantly younger (*p* = 0.02). Surgical correction was performed in 95 cases (77.2%), 14 of them laparoscopically, with a 35.7% conversion rate. Eight cases (6.5%) were managed conservatively. Overall, outcomes were favorable. SH is an infrequent pediatric condition that, based on the synthesized literature, predominantly affects males. The published cases suggest two main clinical phenotypes: a congenital form, often linked to ipsilateral UDT, and an acquired form, typically resulting from trauma. Analysis of the reported data indicates a higher risk of incarceration in early childhood. Surgical treatment is the most frequently reported approach with generally favorable outcomes, whereas the evidence for conservative management remains limited. This comprehensive review highlights the dual nature of pediatric SH and underscores the need for a high index of suspicion in relevant clinical scenarios.

## Simple Summary

Spigelian hernia is a rare condition in children that appears through a natural weak point in the abdominal wall. Some cases are present at birth and may be related to an undescended testicle, while others happen after trauma, such as a bicycle accident. This study reviews all known cases of Spigelian hernia in children described in the medical literature. It found that most of the affected children were boys, and some had complications like intestinal blockage. Early diagnosis and treatment are crucial, especially in infants and younger children. Most children were treated with surgery and recovered well. However, a few mild cases resolved on their own without surgery. This study will help doctors recognize this unusual condition and understand when urgent care or surgery may be needed. It also highlights the importance of thoroughly examining children after blunt abdominal trauma to avoid missing a Spigelian hernia or other serious internal injuries.

## 1. Introduction

Abdominal wall hernias are one of the most common pediatric surgical pathologies worldwide. However, the incidence rate of the different types of hernias is highly variable. While inguinal and umbilical hernias are frequent, other types, such as femoral or Spigelian hernias (SH), are highly infrequent [1,2,3,4,5].

## 2. Anatomy and Embryology of the Semilunaris Line (Spigelian Line)

During embryonic development, the primitive mesoderm formed during gastrulation differentiates into splanchnic mesoderm —which gives rise to the visceral peritoneum and the connective tissue of the gut and associated organs— and somatic mesoderm, which contributes to the parietal peritoneum and the formation of the abdominal wall. Between the fourth and tenth weeks of gestation, myocytes migrate bilaterally from the paravertebral regions to form the muscular and aponeurotic layers of the anterior abdominal wall, completing their fusion and establishing the linea alba by approximately week ten. In the following weeks, these layers gain strength, and the superficial fascia undergoes complete differentiation [6,7]. This process is often associated with congenital points of weakness, such as the linea alba (where epigastric hernias occur) or the umbilicus (where umbilical hernias develop).

The semilunaris line, *linea semilunaris* or Spigelian line (SL) was first described by Adriaan van den Spiegel (1578–1625) as the region of the anterior abdominal wall formed by the transition from muscle to aponeurosis of the *transversus abdominis* muscle. It is a structure located on the lateral margin of the *rectus abdominis* muscle. It extends from the costal margin near the ninth costal cartilage down to the pubic tubercle, following a curved, concave line along the lateral border of the rectus abdominis muscle on the anterior abdominal wall. It is considered a point of fascial weakness [1,2,3]. Although the Spigelian line partially overlaps with the lateral margin of the *rectus abdominis* muscle, recent studies suggest that the anatomic boundaries of these two structures are different [8].

**Figure 1:**
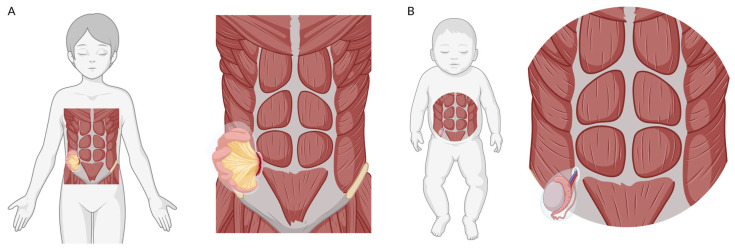
Graphic representation of the two main subtypes of Spigelian hernia in the pediatric age group. (**A**) Acquired (traumatic) right Spigelian hernia. (**B**) Spigelian hernia in the context of Spigelian-Cryptorchidism Syndrome. Created using BioRender. Arredondo Montero, J. (2025) https://BioRender.com/a53g564.

SL is an infrequent but well-described site where hernias can occur. SHs are interparietal defects that occur along the SL, typically deep into the external oblique muscle [4]. For this reason, they are referred to as interparietal hernias (abdominal hernias in which the hernial sac is situated between the layers of the abdominal wall, specifically between the fascias or muscles, rather than completely protruding through them). Regarding pediatric SH, two variants have been described: congenital SH and acquired SH. Figure 1 presents a schematic diagram of the two subtypes of SH described in the pediatric population (congenital and acquired) and their distinguishing characteristics. This review aims to synthesize the published literature on pediatric SH to characterize its epidemiology, clinical presentation, management, and outcomes.

## 3. Methods

We conducted a comprehensive review of the literature on pediatric Spigelian hernia. Eligible studies were identified by searching the primary existing medical bibliography databases (PubMed, Web of Science, Scopus, and Ovid). Supplementary File S1 shows the inclusion and exclusion criteria. JAM and MRJ selected articles using the Covidence^®^ tool. The search results were imported into the platform, and both authors independently screened the articles. Disagreements were resolved by consensus.

A pooled analysis of published case-level data was performed. Regarding descriptive statistics, the median and interquartile range were used for quantitative variables, and proportions were used for categorical variables. The Shapiro–Wilk test was applied to assess the normality of quantitative variables. Homogeneity of variances was evaluated using Levene’s test. Kernel density estimations were performed using an Epanechnikov kernel. These curves represent smoothed distributions of age (in years), and the *Y*-axis is expressed in units of density per year (1/year), such that the total area under each curve equals 1. Mann–Whitney U, Kruskal–Wallis, and Fisher’s exact tests were used to compare sociodemographic and clinical variables between groups. Spearman’s and Pearson’s correlation analyses were used to assess the degree of correlation between variables, while Cramér’s V was applied to evaluate the degree of association among the variables. A *p*-value of <0.05 (two-tailed) was considered statistically significant. All statistical analyses were conducted using STATA 19 (StataCorp; Stata Statistical Software: Release 19; College Station, TX, USA: StataCorp LLC). Supplementary File S2 provides the complete database with all analyzable data extracted from the included articles and primary sources.

## 4. Results

### 4.1. Main Characteristics of the Included Studies, and the Sociodemographic Features of the Patients

The literature review identified 82 indexed publications by 79 first authors [9,10,11,12,13,14,15,16,17,18,19,20,21,22,23,24,25,26,27,28,29,30,31,32,33,34,35,36,37,38,39,40,41,42,43,44,45,46,47,48,49,50,51,52,53,54,55,56,57,58,59,60,61,62,63,64,65,66,67,68,69,70,71,72,73,74,75,76,77,78,79,80,81,82,83,84,85,86,87,88,89,90]. Table 1 summarizes all of the published cases of Spigelian hernia (SH) described in the pediatric population to date.

The distribution of publications by country was as follows: USA (n = 23), India (n = 10), Italy (n = 6), Turkey (n = 5), Spain (n = 4), United Kingdom (n = 4), Australia (n = 4), Greece (n = 3), Israel (n = 3), Japan (n = 3), New Zealand (n = 2), and Saudi Arabia (n = 2). Algeria, Ireland, Norway, Germany, Puerto Rico, Canada, Ghana, and Tunisia each contributed one publication. The geographic origin of five publications could not be determined. A total of 123 patients were reported. Of these, 106 were male (86.2%) and 17 were female (13.8%). Age ranged from birth to 21 years (median = 3, interquartile range = 0.25–9 years). Forty-six patients were aged one year or younger. Notably, this age distribution is markedly bimodal, reflecting a peak in early infancy (corresponding primarily to congenital cases) and a second peak in late childhood (often associated with traumatic etiologies).

### 4.2. Characteristics of the Spigelian Hernia

Forty-seven patients (38.2%) had a left-sided SH, fifty-six (45.5%) had a right-sided SH, and thirteen (10.6%) had a bilateral SH. In seven cases (5.7%), laterality was not explicitly reported. Concerning SH measurements, 55 cases reported numerical values, of which 15 provided two independent values in cm (major and minor axes) and 40 provided a single value in cm (corresponding to the major axis). Significant heterogeneity was identified in the methodology used to determine the measurements. Some authors utilized radiological or intraoperative measurements of the fascial defect (ring), while others, such as Fraser et al., measured the diameter of the cutaneous bulge [40]. Certain researchers (Vaos et al., Inan et al., and Upasani et al.) provided both characterizations, noting a substantially larger size of the cutaneous protrusions compared to the actual size of the fascial defect [44,56,65]. Meanwhile, other authors, such as Bilici et al. and Okumuş et al. [87], provided a range of measurements for the defect [60,87]. For the statistical analysis of these patients, the central value of the range was used as the imputed value.

For cases with at least one linear dimension available (n = 55), the median (interquartile range) was 2.4 (1.5–4.0) cm. For cases reported with two measurements (n = 15), values were converted into surface area (cm^2^), yielding a median (interquartile range) of 15 (4.32–21) cm^2^. No statistically significant differences were found in the size of fascial defects between patients with undescended testis (UDT) and those without UDT (*p* = 0.96), nor between patients with traumatic etiology and those without traumatic etiology (*p* = 0.45). No differences in fascial size were observed based on age (Spearman, *p* = 0.93) or sex (*p* = 0.68). Similarly, no statistically significant differences were found concerning laterality (Kruskal–Wallis, *p* = 0.65).

### 4.3. Spigelian Hernia Etiology and Associations

Regarding etiology, 45 out of 123 cases (36.6%) were classified as acquired or traumatic. Specifically, thirty cases (24.4%) were caused by handlebar trauma (29 from bicycles and one from a motorcycle), three cases (2.4%) were related to prior surgical procedures, two (1.6%) by road accidents, two (1.6%) by bicycle falls, two (1.6%) by unspecified local trauma, and two (1.6%) by BMX accidents. The remaining cases included one injury from cow goring (0.8%), one from a nail puncture (0.8%), one ATV accident (0.8%), and one instance of unspecified bicycle trauma (0.8%). The remaining cases were either congenital or had no attributable etiology described. No statistically significant differences were found between patients with traumatic and non-traumatic SH based on gender (*p* = 0.57).

Forty-one patients (33.3%) presented with an SH associated with UDT. Concerning laterality, 16 (39%) UDT were left-sided, 15 (36.6%) right-sided, and 10 (24.4%) bilateral. Fisher’s exact test confirmed a statistically significant association between the side of SH and the side of UDT (*p* < 0.0001). When a Cramér’s V analysis was performed between the side of SH and the side of UDT, a very strong association was found (0.83).

Regarding age and the etiology of SH, the median (interquartile range) age of patients with traumatic SH was 9 (7–12) years, while for patients with non-traumatic SH, it was 0.68 (0.08–4) years (*p* < 0.0001). Similarly, patients with SH and associated UDT had a median (interquartile range) age of 0.25 (0.04–1.08) years, compared to a median (interquartile range) age of 8 (2.5–11) years for patients without UDT (*p* < 0.0001).

**Figure 2:**
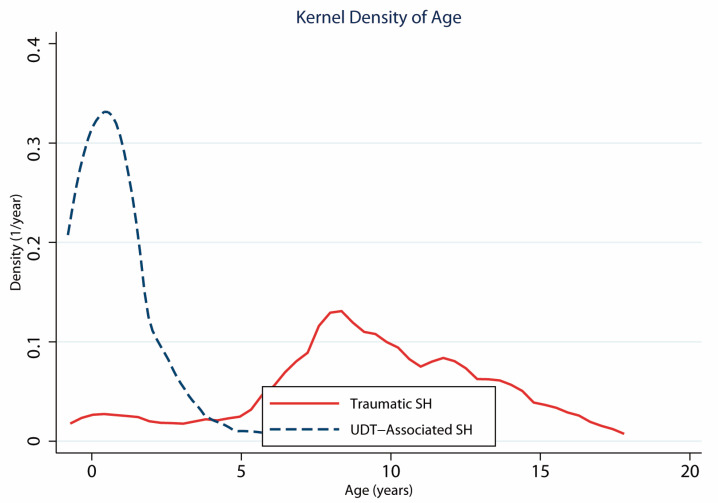
Kernel density estimates of patient age at diagnosis for Spigelian hernia (SH) cases associated with undescended testis (UDT, dashed navy line) and trauma-related cases (solid red line). The *y*-axis represents the probability density per year (1/year), and the total area under each curve sums to 1, allowing for direct interpretation as the proportion of cases across age intervals. The plot reveals a bimodal distribution, with a marked concentration of UDT-associated SH during the first year of life—supporting its likely congenital origin—and a second distinct peak for trauma-related SH between 7 and 9 years of age, consistent with the typical age range for handlebar injuries.

Figure 2 displays histograms and kernel density estimates for both etiologies, revealing a peak incidence of traumatic SH between 7 and 9 years of age, with a marked concentration of SH cases related to UDT occurring predominantly during the first year of life. In this analysis, the presence of UDT was used as a reference for congenital SH in both epidemiological and analytical terms, although a perfect equivalence cannot be assumed.

### 4.4. Complications Associated with Spigelian Hernia

In total, 15 out of 123 patients (12.2%) were reported to have some hernia incarceration or strangulation. These 15 patients were significantly younger than the group of patients without hernia incarceration, 0.15 (0.08-7) years vs. 4 (0.5-9) years (*p* = 0.02), but no gender differences were seen (*p* = 0.63). Additionally, no differences were found in the hernia incarceration rate between the UDT group and the non-UDT group (*p* = 0.37), nor between the traumatic and non-traumatic SH groups (*p* = 0.25). Seven visceral injuries (5.7%) were described, all of them in patients with traumatic SH and attributable to the injury mechanism that caused the SH (*p* = 0.001). A total of two deaths associated with SH incarceration have been reported in the pediatric population [9,80]. A.H. Al-Salem reported an additional death from sepsis, apparently unrelated to SH [37]. 

### 4.5. Spigelian Hernia Diagnosis

Regarding the most commonly used imaging modalities for the diagnosis of SH, plain abdominal X-rays were reported in ten patients (8.1%), with most cases corresponding to traumatic or incarcerated/strangulated SH. In 32 patients (26%), ultrasound scan (US) was reported as the imaging modality used, and in 20 patients (16.3%), computed tomography (CT) was reported, with the vast majority being trauma cases. Several authors reported using two or more imaging modalities.

### 4.6. Spigelian Hernia Treatment

In 95 patients (77.2%), surgical correction of the defect was reported, and in at least 15 of them (15.8%), it was performed urgently. The treatment provided was not detailed in nine studies (9.6%). Of the surgically treated patients, 14 (14.7%) were approached laparoscopically. Of these, eight (57.1%) were repaired entirely laparoscopically, one (7.1%) was laparoscopically explored but not repaired, and five (35.7%) required conversion to open surgery.

In most cases, primary repair of the defect was performed. Few authors reported the use of mesh for the repair of pediatric SH. Singal et al. [55] reported using a VYPRO™ mesh (Ethicon, Somerville, NJ, USA), and Fascetti-Leon et al. reported using a Vicryl™ mesh (Ethicon, Somerville, NJ, USA) [51].

Eight patients (6.5%) were managed conservatively. Of these, three (37.5%) had a complete spontaneous resolution, three (37.5%) had a partial resolution (these three cases were traumatic SH with a limited follow-up period), one (12.5%) patient died, and one (12.5%) had no reported follow-up.

Table 2 summarizes the review’s key sociodemographic and clinical findings, offering a concise overview to facilitate interpretation.

### 4.7. Spigelian Hernia: Overall Outcomes and Surgery-Related Complications

Ninety-five patients (77.2%) were reported as having a favorable outcome, with a highly variable follow-up period. Nineteen (15.4%) had no data on their follow-up and outcome. Two patients (1.6%) died due to SH strangulation. Although the number of patients with a favorable outcome (n = 95) coincidentally matches the number of surgically treated patients, this group is composed differently. It includes patients who were successfully managed conservatively and excludes surgical patients who experienced complications.

Concerning post-surgical complications, reported in a total of five patients (4.1%), one patient (0.8%) experienced persistent postoperative pain, and one (0.8%) had abdominal wall laxity. Furthermore, three patients (2.4%) with SH associated with UDT developed a scrotal abscess, two of whom (1.6%) subsequently developed secondary testicular atrophy.

Additionally, one patient died from sepsis unrelated to the SH (0.8%), and another developed a pancreatic pseudocyst as a complication of the initial trauma rather than of the hernia surgery itself (0.8%).

## 5. Discussion

The reported cases demonstrate a broad geographical distribution, and no consistent pattern was identified. We found a clear predominance of this pathology in males (86.2%) and a slight predominance of right-sided SH over left-sided SH (45.5% vs. 38.2%), with a relatively high proportion of bilateral SH cases (10.6%). A significant variability has been identified in the way fascial defects are characterized, making it challenging to extrapolate information on this aspect.

Trauma-related SH accounts for 36.6% of reported cases in the literature. This type predominantly occurs in the age range of 7–9 years, with no sex predominance. Trauma-related SH has been associated with various injury mechanisms, with a clear predominance of activities involving bicycles, motorcycles, and BMX bikes. The most common mechanisms are sudden deceleration and a blunt impact from the handlebar on the SL. In these cases, visceral injuries of various kinds have been documented. Therefore, in the presence of trauma with associated SH (or handlebar trauma), one must assume a high-energy injury mechanism and conduct a thorough screening for underlying injuries that could compromise life and whose diagnostic delay may be associated with morbidity and mortality. It is also relevant to mention that several cases have been reported as ‘handlebar hernia’ in a nonspecific manner. In our literature review, we were able to categorize some of these as SH (based on anatomical description or CT images). Still, others corresponded to anatomical areas of the abdominal wall different from SL. For this reason, we consider it essential that future authors report the anatomy of the lesions accurately and in detail, as there may be diagnostic, therapeutic, and prognostic variations between SH and other parietal lesions.

On the other hand, SH associated with UDT represents 33.3% of pediatric SH reported cases. In most instances, this is assumed to be a congenital hernia, with a strong correlation between the side of the UDT and the hernia. The high number of patients with associated anterior wall defects, ranging from non-formed inguinal canals to hernias, is striking. For example, Silberstein et al. [32] reported an absence of inguinal canal formation with associated muscle atrophy. Ostlie et al. [35], V. Raveenthiran [43], Bilici et al. [60], and Parihar et al. [63] reported similar findings. A.H. Al-Salem [37], Ostlie et al. [35], Bilici et al. [60], and Parihar et al. [63] reported an absence of *Gubernaculum testis* formation. More recent authors, such as Gonuguntla et al. [85], reported similar findings. In general, when muscular alterations have been reported, they have been described regarding the internal oblique and *transversus abdominis* muscles and have been described as hypoplastic, thinned out, or even absent (Komura et al., Singal et al., Inan et al.) [29,55,56]. Komura et al. reported in 1994 [29] the histology of the muscles adjacent to the affected area in SH, demonstrating atrophy with fat infiltration and interstitial fibrosis. This dichotomy in etiology likely reflects distinct pathophysiological processes. Traumatic hernias represent acute disruptions of otherwise healthy fascial tissue, which may retain intrinsic healing potential, justifying a trial of conservative management in highly selected cases. In contrast, congenital hernias, particularly those associated with UDT and reported muscular hypoplasia, may stem from a primary developmental defect in the abdominal wall, rendering spontaneous closure biologically less plausible. This fundamental distinction provides a strong rationale for recommending early surgical repair for congenital defects, even if asymptomatic.

Lastly, it is relevant to consider that some authors have proposed that UDT and SH may constitute a pathological spectrum encompassed under the term “Spigelian-Cryptorchidism Syndrome”. It has also been suggested that this could be a variant of Prune Belly syndrome. However, we have not found a consistent pattern of visceral, cardiac, genitourinary, or musculoskeletal malformations in the reported patients.

Regarding complications, a relatively high percentage of patients (12.2%) experienced hernia incarceration or strangulation, with this complication predominantly occurring in younger patients. This percentage, significantly higher than other hernia-related conditions such as umbilical or epigastric hernias, should be considered when recommending prompt correction of the defect, especially during early childhood.

Diagnosing this condition has traditionally been considered challenging due to the limited clinical expression of the defect (secondary to its intraparietal location). Given that pediatric patients have a thinner anterior abdominal wall due to less muscular development and a reduced adipose layer, these hernias are potentially easier to identify clinically. However, familiarity with the anatomy of the abdominal wall and a high index of suspicion are essential for an accurate diagnosis. Several cases of misdiagnosis and diagnostic confusion with other pediatric abdominal wall defects, such as inguinal hernia, have been reported [45].

Concerning radiology tests, this review highlights the use of different imaging modalities for this condition, with a predominance of plain abdominal X-rays and CT scans in urgent or trauma scenarios and an increasing use of ultrasound in elective settings, particularly in recent years. It is important to emphasize that SH is an uncommon diagnosis in the pediatric population, and radiological studies should be performed in a targeted manner by expert radiologists to avoid diagnostic errors.

The most widely accepted and utilized treatment approach in the existing literature is open surgery. Laparoscopy has been employed in selective cases, although it is associated with a relatively high conversion rate. Conservative management is scarcely documented for this condition but has proven to be effective in some patients. From the perspective of biological plausibility and surgical safety, we believe that conservative management should be limited to minor fascial defects in healthy patients with a traumatic etiology who are asymptomatic and show no herniated contents on imaging studies. Likewise, we consider that these patients require close clinical and ultrasound follow-up.

Concerning the use of mesh, although the evidence is limited, given the favorable healing profile of the pediatric population and the excellent outcomes reported in cases with primary repair, the routine use of mesh for the surgical correction of this condition is not justified.

Regarding the surgical prognosis of UDT specifically in this context, the majority of authors have reported the successful performance of primary orchidopexy in a single stage. We attribute this to the fact that the hernial sac allows for progressive elongation of the spermatic vessels and the vas deferens, in contrast to purely intra-abdominal testes. Concerning medium and long-term results, most published cases report a favorable outcome, but with a variable follow-up period. However, in many cases, follow-up is limited or not reported at all. Once again, it is essential to address this aspect to better understand this condition in prognostic terms.

This review has several significant strengths, including a comprehensive analysis of the existing literature and its statistical approach, with findings that are relevant to understanding the disease. However, it also presents notable limitations. First, although our search strategy, screening process, and data extraction closely mirrored those of a systematic review, the nature of the research question did not align with a classical PICO framework, and no comparator or intervention was predefined. In addition, the evidence base—composed mainly of case reports and small case series—lacked the methodological consistency necessary to allow for risk-of-bias assessment. For these reasons, we deliberately chose not to frame this work as a systematic review to avoid overstating its methodological rigor and reproducibility.

Second, while no aggregate-level meta-analysis was feasible, we conducted a pooled analysis of case-level data extracted from published studies. This approach allowed for granular, patient-level statistical analyses, but comes with inherent limitations: lack of access to raw data, inconsistent reporting, potential duplication of cases, and the inability to verify outcomes or adjust for confounding variables using multivariable modeling. Moreover, as the data were reconstructed from published reports rather than obtained directly from investigators, the study does not meet the methodological standards of an individual patient data meta-analysis (IPD-MA), precluding adherence to PRISMA-IPD requirements such as data verification and integrity checks. Also, while nonparametric tests were a reasonable choice given the data structure, the application of inferential statistics is constrained by the heterogeneity and historical breadth of the published reports. The compiled cases represent a convenience sample spanning almost a century and are subject to profound, unquantifiable biases that invalidate the core assumptions of hypothesis testing. These include publication bias (whereby more severe or unusual cases are more likely to be reported), notification bias (inconsistent reporting of variables across studies), and significant temporal bias due to evolving clinical and diagnostic practices over nine decades. Consequently, any inferential findings, such as *p*-values, should be interpreted as mathematical properties of this specific dataset rather than robust evidence of a clinical association, and the study’s conclusions must remain within a descriptive and exploratory framework.

Third, several of the included reports were incomplete or lacked uniformity in the variables provided, further restricting the granularity and consistency of certain subgroup analyses. Lastly, while our statistical inferences are exploratory and aimed at hypothesis generation, they must be interpreted with caution due to small subgroup sizes, a large number of comparisons, and the increased risk of type I error and residual confounding.

Future research directions should aim to refine the classification of pediatric Spigelian hernia into traumatic and non-traumatic subtypes, ideally distinguishing congenital forms more explicitly. This would enable more meaningful comparisons regarding associated anomalies—such as UDT—and complication rates, particularly strangulation. Although our dataset reflects a high frequency of UDT among non-traumatic cases, the absence of consistent diagnostic criteria for congenital SH and the lack of universal association with UDT precluded a definitive stratification in the present study. Similarly, while the link between SH, UDT, and syndromic entities, such as Prune Belly syndrome or spinal dysraphism, is anatomically plausible—potentially mediated by reduced intra-abdominal pressure—our ability to explore this hypothesis was constrained by the small number and heterogeneity of patients with complex syndromic presentations. Future collaborative efforts with larger case series and more granular reporting could allow for robust subgroup analyses and help clarify the developmental and pathophysiological underpinnings of this rare condition.

In conclusion, SH is an uncommon pediatric condition that predominantly affects males. It may present congenitally—often associated with ipsilateral UDT—or be acquired, typically following blunt abdominal trauma involving the SL, most frequently from bicycle handlebar injuries. The risk of incarceration is relatively high, particularly during early childhood, and most reported cases have been managed surgically with favorable outcomes. Although evidence for successful nonoperative management exists, it remains limited to carefully selected trauma-related cases.

From a practical standpoint, this review highlights key diagnostic and management considerations for pediatric surgeons. Spigelian hernia should be suspected in infants presenting with an ipsilateral undescended testis—particularly when the inguinal canal is absent or non-palpable—as well as in school-age children with blunt abdominal trauma, especially from bicycle handlebars. Early use of targeted ultrasound by experienced radiologists is recommended to confirm the diagnosis, given the intraparietal location of the defect and its potential to be overlooked. Due to the relatively high risk of incarceration in young children, prompt surgical repair is the most prudent approach, even in asymptomatic congenital cases, particularly when associated with an undescended testis. The reported mortality, although scarce, further supports this recommendation—along with the low likelihood of spontaneous testicular descent and the potential underlying malformative or genetic muscular defect that may hinder spontaneous fascial closure. Conservative management may be cautiously considered in select trauma-related cases with minor, asymptomatic defects and no herniated contents, but requires close clinical and radiologic follow-up. These findings support the need for heightened clinical suspicion and timely intervention to optimize outcomes in this rare but potentially serious condition.

## Figures and Tables

**Table 1:** Previously reported cases of pediatric Spigelian hernia in the medical literature.

Author	Country	Patient’s Age	Patient’s Sex	Laterality	Associated Malformations/Anomalies	Complications Associated with SH	Relevant Background	Defect Size	Radiological Studies	Treatment	Surgical Approach/Findings	Outcome
A.J. Scopinaro (1935) [9]	-	6 d	Male	-	-	Strangulation	-	-	-	-	-	Death
Hurwitt et al. (1955) [10]	-	8 y	Male	Bilateral	-	-	-	Right side: 2.6 cm	-	-	-	-
R.M. Landry (1956) [11]	-	14 y	Male	Left	-	-	Previous local trauma	4 cm	-	-	-	-
N.H. Isaacson (1956) [12] *	USA	3 y	Male	Right	-	-	Nail puncture	-	-	-	-	-
T.H. Wilson (1959) [13]	USA	2.5 y	Male	Left	-	-	Previous local trauma (automobile)	-	Plain abdominal X-ray: Traumatic hernia of the abdominal wall	Surgical	ESR 6 days after the accident Hernial sac containing omentum and transverse colon. Repair: Interrupted silk suture	-
G.R. Roberts (1964) [14] *****	UK	9 y	Male	Left	-	-	Previous local trauma (bicycle handlebar)	7 × 3 cm	-	Surgical	Repair: Interrupted catgut suture	Favorable (6 m follow-up)
S. Bertelsen (1966) [15] *	-	9 y	Male	Right	-	-	-	2 × 2 cm	-	-	-	Favorable
Hurlbut et al. (1967) [16]	USA	8 y	Male	Right	-	-	Previous local trauma.	3 × 5 cm	Plain abdominal X-ray: Gas-filled small bowel loops compatible with an incarcerated hernia	Surgical	ESR	Favorable
Graivier et al. (1967–1988) [17,18,19]	USA	(1) 10 m (2) 6 m (3) 6 m (4) 9 m (5) 15 y (6) 17 y (7) 15 m (8) 4 y (9) 10 d	(1) Male (2) Male (3) Male (4) Male (5) Female (6) Female (7) Female (8) Female (9) Female	(1) Bilateral (2) Right (3) Right (4) Left (5) Right (6) Bilateral (7) Bilateral (8) Left (9) Left	(1) Umbilical Hernia and bilateral indirect Inguinal hernias	-	-	(1) 2 × 5 cm (left) 1 × 3 cm (right)	-	Surgical	(1–8): ESR (9): Lost of follow-up before surgery. Hernial sacs: Resected or reduced. Repair: Interrupted silk suture	(1-8): Favorable. No recurrences (with a maximum follow-up of 19 y) (9): Lost of follow-up before surgery.
Herbert et al. (1973) [20]	Canada	7 y	Male	Left	-	-	Previous local trauma (bicycle handlebar)	4 cm (bulge) 3 cm (defect)	Plain Abdominal X-ray: Acute gastric dilatation	Surgical	ESR 3 weeks after the accident. SH repair: Interrupted 2-0 polyglycolic acid suture	Favorable
Constantino et al. (1974) [21]	-	8 y	Male	Left	-	Strangulation	-	-	-	-	-	Favorable
Atiemo et al. (1974) [22] *****	Ghana	6 y	Male	Left	Splenic enlargement (incidental finding)	-	Previous local trauma (cow goring)	-	-	Surgical	ESR 6 days after the accident. Full abdominal wall hernia. Primary repair	Favorable
T.J. Houlihan (1976) [23]	USA	21 y	Male	Bilateral	-	-	-	-	-	-	ESR	Favorable
Jarvis et al. (1977) [24]	USA	13 y	Female	Left	-	-	-	3 cm	-	Surgical	SH. Peritoneal sac present	-
Bar-Maor et al. (1989) [25]	Israel	(1) 5 y (2) 3 m	(1) Female (2) Male	(1) Right (2) Left	(2) Left Bochdalek hernia	-	(1) Abdominal blunt trauma (road accident) (2) Previous abdominal surgery with forceful stretching of the abdominal wall (Bochdalek hernia)	(1) 4 cm (2) 1 cm	-	(1) Conservative (spontaneous resolution). (2) Surgical	ESR. SH	Favorable
J.C. Mitchiner (1990) [26] *****	USA	7 y	Male	Left	-	Incarceration	Previous local trauma (bicycle handlebar)	6 cm	CT (oral and intravenous contrast media): Rent in the anterior abdominal wall with a loop of small bowel exteriorized in the subcutaneous tissue	Surgical (urgent)	Three feet of viable small bowel were found in the subcutaneous tissue protruding through a significant fascial defect. Reduction and primary repair	Favorable (4 m follow-up)
Damschen et al. (1994) [27] *****	USA	5 y	Male	Right	-	-	Previous local trauma (bicycle handlebar)	-	-	Surgical	Urgent surgery: SH. Small bowel herniating through the muscle layers. Repair: Interrupted polyglycolic acid suture	Lost to follow-up (unknown)
G. Kubalak (1994) [28] *****	USA	8 y	Male	Right	-	-	Previous local trauma (bicycle handlebar)	6 cm	-	-	-	Favorable (2 y follow-up)
Komura et al. (1994) [29]	Japan	(1) 6 m (2) 8 m (3) 3 y	(1) Female (2) Female (3) Male	(1) Right (2) Right (3) Left	(1) Ipsilateral mediastinal neuroblastoma (10–12th IC spaces)—Incidental diagnosis of tumor during ultrasound examination of the SH (2) Ipsilateral mediastinal neuroblastoma (9–10th IC spaces)	-	(2) Previous tumor extirpation (1 week before)	(1) 5 × 3 cm (2) 4 cm (3) 7 cm	(3) US: Hernial orifice 4 × 3 cm. Thin muscular layer in the area (3 mm). MRI: Fatty infiltration of internal oblique/transverse muscles	(1) Surgical (2) Conservative (3) Surgical	(1) SH. Peritoneal sac present. Primary repair (mattress 3-0 silk sutures) ** (3) SH. Primary repair (mattress 2-0 silk sutures) **	(1) Favorable (5 y follow-up) (2) Favorable (spontaneous resolution) *** (3) Favorable (2 m follow-up)
Pul et al. (1994) [30]	Turkey	(1) 18 m (2) 2.5 m	(1) Male (2) Male	(1) Right (2) Right	(1) Right UDT (2) Right indirect inguinal hernia.	-	-	(1) 7 × 7 cm (2) 4 × 4 cm		(1) Surgical	(1) SH. Small ring defect (1 × 1 cm). Peritoneal sac present and excised. Primary repair (2) SH. Small ring defect (2 cm). Peritoneal sac present and excised. Primary repair	(1) Favorable (2) Favorable
J.E. Wright (1994) [31]	Australia	(1) 19 m (2) 5 y (3) 7 y	(1) Male (2) Male (3) Male	(1) Right (2) Right (3) Left	-	-	-	-	-	(3) Surgical	(3) SH. Peritoneal sac present (not excised). Two-layer repair	(3) Favorable (8 y follow-up)
Silberstein et al. (1996) [32]	Australia	(1) Newborn (2) Newborn	(1) Male (2) Male	(1) Left (2) Right	(1) Contralateral generalized abdominal musculature weakness. Left UDT (2) Right UDT	-	-	(2) 3 cm	-	(1) Surgical (10 w of age) (2) Surgical (4.5 m of age)	(1) SH. Peritoneal sac present, containing the left testis (small in size, without testicular–epididymal dissociation). Absence of the inguinal canal. Primary repair and orchidopexy (2) SH. The internal oblique and transversalis muscles were poorly developed. Peritoneal sac present, containing the right testis. Absence of the inguinal canal. Primary repair and orchidopexy	-
Iuchtman et al. (1997) [33]	Israel	7 y	Male	-	-	-	Previous local trauma (bicycle)	-	US: Normal	Surgical	Urgent surgery: SH. Peritoneal protrusion (intact peritoneum). Primary repair without mesh	Favorable
Pérez et al. (1998) [34] *****	USA	11 y	Male	Left	-	-	Previous local trauma (bicycle handlebar)	-	-	Surgical	Urgent surgery: SH. Peritoneal tearing. Repair in layers	Favorable
Ostlie et al. (1998) [35]	USA	Newborn	Male	Right	Right UDT. The right testis was palpable in the hernial sac	-	-	1.5 cm	-	Surgical	SH. Thinning of the layers. Absence of the inguinal canal. Peritoneal sac containing the right testis. Primary repair (absorbable sutures) and orchidopexy	-
Kubota et al. (1999) [36] *****	Japan	9 y	Male	Right	-	-	Previous local trauma (bicycle handlebar)	4 cm	CT: Rent in the abdominal wall through which intestinal loops protruded into the subcutaneous space	Surgical	SH. Peritoneal rupture. Primary repair	Favorable
A.H. Al-Salem (2000) [37]	Saudi Arabia	(1) 3 m (2) Newborn	(1) Male (2) Male	(1) Left (2) Left	(1) Left UDT (2) Micrognathia, cleft palate, malformed ears, right clubfoot, malformed left lower limb, left UDT. Left testis palpable on the hernial sac. (Insulin-dependent diabetic mother)	-	-	(1) 5 cm	(1) US: Normal	(1) Surgical	(1) SH. Peritoneal sac present containing the left testis (small in size, without testicular–epididymal dissociation) and sigmoid colon. Primary repair and orchidopexy	(1) Favorable (2.5 y follow-up) (2) Died before surgery (sepsis, not related to SH)
J.J. White (2002) [38]	USA	1 m	Female	Right	Bilateral inguinal hernia	Incarceration/small bowel obstruction	SH appeared in the immediate postoperative period after bilateral inguinal hernia repair	1.5 cm (ring)	Plain abdominal X-ray: Partial small bowel obstruction US: Bowel in the mass, under the skin	Surgical	SH. Reduction in the small bowel, primary repair	Favorable
Losanoff et al. (2002) [39]	USA	12 y	Male	Right	-	Omentum incarceration mimicking acute appendicitis	-	1.5 cm	Plain chest and abdominal X-rays: Normal	Surgical (urgent)	Normal appendix. SH with incarcerated and infarcted omentum. Resection, primary repair	Favorable (6 m follow-up)
Fraser et al. (2002) [40] *****	UK	11 y	Male	Right	-	Inguinal hematoma	Previous local trauma (bicycle handlebar)	12 × 8 cm (bulge)	US: Disruption of the muscle layers of the abdominal wall with bowel and free fluid lateral to the rectus muscle. Plain abdominal X-ray (lateral): A collection of gas was visible in the anterior abdominal wall	Surgical	Traumatic hernia involving all layers of the lower abdominal wall above the inguinal canal. Primary repair (absorbable suture)	Favorable
Levy et al. (2003) [41]	Israel	(1) 1 m (2) 5 w	(1) Male (2) Male	(1) Bilateral (2) Left	(1) Bilateral UDT (2) Left UDT	(1) Incarceration	-	(2) 5 cm	(1) US (right side): SH containing incarcerated bowel loops and the undescended right testis. US (left side): small SH containing the undescended left testis (2) US: Left SH containing bowel loops and the undescended left testis	(1) Right side: Surgical (urgent). Left side: Surgical (deferred, with 10 m). (2) Surgical (deferred, with 2 m)	(1) Right side: SH. Small bowel reduction, primary repair, right orchidopexy. Left side: SH primary repair, left orchidopexy. (2) SH. Peritoneal sac present, containing small bowel and left testis. Reduction, primary repair, and orchidopexy	Favorable
Goliath et al. (2004) [42] *****	USA	11 y	Male	Right	-	-	Previous local trauma (bicycle handlebar)	-	CT: Intestinal loops protruding through a defect in the abdominal wall into the subcutaneous space	Surgical	Defect throughout his entire abdominal wall, including the fascia, muscular layers, and peritoneum, with bowel protruding into the subcutaneous space, leaving his skin and intra-abdominal organs completely intact. Primary repair	Favorable
V. Raveenthiran (2005) [43]	India	2 d	Male	Right	Imperforated anus. Bilateral UDT. Hypoplastic scrotum. Left inguinal hernia (noted with 1 m of life), umbilical hernia (noted with 2 m of life)	-	-	5 × 5 cm	US: Bowel loops in the intermuscular plane at the site of the SH	Surgical (deferred, with 13 m)	SH. Peritoneal sac present, containing the right testis. Absence of the inguinal canal	Favorable
Vaos et al. (2005) [44]	Greece	(1) 20 m (2) 8 m	(1) Male (2) Male	(1) Left (2) Right	-	(1) Strangulation (2) Strangulation	-	(1) 2.5 cm (bulge), 2 cm (ring) (2) 2 cm (bulge), 1 cm (ring)	(1) Plain abdominal X-ray: Several gas-fluid levels in the small bowel	(1) Surgical (urgent) (2) Surgical (urgent)	(1) SH. Peritoneal sac present, containing incarcerated viable small bowel loops and infarcted omentum. Omentectomy, reduction, primary repair (2) SH. Peritoneal sac present, containing incarcerated viable small bowel loops. Reduction, primary repair	(1) Favorable (12 m follow-up) (2) Favorable (6 m follow-up)
Torres de Aguirre (2005) [45]	Spain	(1) Newborn (2) Infant (5 w)	(1) Male (2) Male	(1) Right (2) Bilateral	(1) Right UDT (2) Bilateral UDT	(1) Incarceration (2) Right incarceration	-	-	(1) US and plain abdominal x-ray: Incarcerated right inguinal hernia	(1) Surgical (urgent) (2) Surgical (urgent)	(1) SH. Peritoneal sac present, containing incarcerated small bowel and right testis. Reduction, primary repair, and orchidopexy (2) Bilateral SH. Right: Peritoneal sac present, containing incarcerated viable small bowel loops. Reduction, primary repair. Left: peritoneal sac present containing a hypoplastic left testis with epididymal–testicular dissociation. Orchiectomy and primary repair	Favorable
Durham et al. (2006) [46]	USA	(1) 8 m (2) 13 m (3) 14 m **** (4) 2 m ****	(1) Male (2) Male (3) Male (4) Male	(1) Left (2) Bilateral (3) Bilateral (4) Right	(1) Left UDT (2) Bilateral UDT (3) Bilateral UDT (4) Bilateral UDT	-	-	-	-	Surgical	(1) SH. Peritoneal sac present, containing the left testis. Orchidopexy and primary repair (2) Bilateral SH. Staged repair: Right side with 13 m and left with 16 m. Peritoneal sac present, containing testes. Orchidopexy and primary repair (absorbable sutures, 8-ply SIS mesh on right side) (3) bilateral SH. Repair. Testicles found in their ipsilateral SH sac. Orchidopexy and primary repair (4) With 13 m: Right intra-abdominal testis: orchidopexy. Right SH: Primary repair. With 16 m: Left inguinal orchidopexy	(1) Complication: Scrotal abscess. Loss of follow-up (2) Complication: Laxity on the right side where the SIS patch was used (3) Favorable (45 m follow-up) (4) Favorable (12 m follow-up)
O’Sullivan et al. (2006) [47]	Ireland	4 m	Male	Left	Left UDT. Hypospadias	-	-	-	-	Surgical	SH. Peritoneal sac present, containing the left testis. Orchidopexy, primary repair.	-
Kumar et al. (2007) [48]	India	3 y	Male	Right	Right UDT	-	-	7 cm (neck of peritoneal sac)	-	Surgical	SH. Peritoneal sac present, containing the right testis. Orchidopexy, primary repair (VYPRO™ mesh)	Favorable
Aksu et al. (2007) [49]	Turkey	4 y	Female	Bilateral	Consanguineous parents. Multiple skeletal anomalies	-	-	6.5 cm (bulges), 3 × 2 cm (right ring) 2 × 1 cm (left ring)	-	Surgical	Bilateral SH. Internal oblique and *transverse abdominis* missing. Peritoneal sac present, containing small bowel loops. Reduction, primary repair (3/0 Vicryl™ sutures)	Favorable (8 m follow-up)
Litton et al. (2008) [50] *****	UK	13 y	Male	Right	-	-	Previous local trauma (bicycle handlebar)		CT: Herniation of bowels. No intra-abdominal injury	Conservative (spontaneous resolution)	-	Favorable (4 m follow-up)
Fascetti-Leon et al. (2009) [51]	Italy	Newborn	Male	Bilateral	Bilateral UDT. Scalp aplasia cutis. Hypertelorism, delayed growth, small head circumference, hypoplasia of the nasal alae, and dentition anomalies	-	-	-	US: Bilateral SH. Bilateral UDT	Surgical	Bilateral SH. Peritoneal sac present, containing testes. Orchidopexy and primary repair (Vicryl™ mesh)	Favorable (12 m follow-up)
Christianakis et al. (2009) [52]	Greece	6 y	Male	Left	-	Inguinoscrotal pain	-	1.5 cm (ring)	-	Surgical	SH. Peritoneal sac present. Primary repair (non-absorbable sutures)	Favorable (8 y follow-up)
Rushfeldt et al. (2010) [53]	Norway	Newborn	Male	Right	Right UDT	Incarceration	-	-	US: SH with a hernial sac between the internal and external oblique muscles (7 mm hernia opening) containing the right testis and a loop of small bowel	Surgical (urgent)	SH. Peritoneal sac present, containing incarcerated viable small bowel loops and right testis. Reduction, orchidopexy, primary repair	Favorable
Vega et al. (2010) [54]	Puerto Rico	9 y	Male	Left	-	-	-	-	-	Surgical	SH. Primary repair	-
Singal et al. (2011) [55]	India	(1) 3 y (2) 3 m	(1) Male (2) Male	(1) Right (2) Left	(1) Right UDT (2) Left UDT. Glanular hypospadias	-	-	(1) 7 cm (ring/neck)	-	Surgical	(1) SH. Thinned out internal oblique. Peritoneal sac present, containing the right testis. Orchidopexy and primary repair (VYPRO™ mesh) (2) SH. Peritoneal sac present, containing the left testis. No inguinal canal or *gubernaculum testis* present. Orchidopexy and primary repair	(1) Favorable (4 y follow-up) (2) Favorable (1 y follow-up)
Inan et al. (2011) [56]	Turkey	26 d	Male	Right	Right UDT	-	-	3 cm (bulge), 2 cm (ring)	US: Fascial plane defect through the *linea* *semilunaris* with herniation of bowel loops between the internal and external oblique muscles. Absence of testis in the right scrotum. No inguinal canal or spermatic cord	Surgical	SH. Thinning of layers. Peritoneal sac present, containing the right testis. Orchidopexy and primary repair	Complication: Scrotal abscess (postoperative day 8) and atrophy of the testis
Lopez et al. (2011) [57,59]	New Zealand	14 y	Male	Left	-	-	Previous local trauma (bicycle handlebar)	-	CT: Herniation of fat and vessels through a defect in the abdominal wall musculature consistent with a diagnosis of SH	Surgical	SH. Laparoscopic repair	-
Yan et al. (2011) [58] *****	Australia	8 y	Male	Right	-	-	Previous local trauma (BMX handlebar)	-	CT: TAWH	Surgical (urgent)	TAWH. Primary repair	Favorable (1 m follow-up)
Bilici et al. (2012) [60]	Turkey	(1) 6 m (2) 1 y (3) 2 y (4) 5 y	(1) Male (2) Male (3) Male (4) Male	2 patients: left 2 patients: right	4 patients: Ipsilateral UDT	4 patients: Abdominal distension	-	1.5 to 2.5 cm	-	Surgical	SH. Peritoneal sac present, containing the ipsilateral testis (all cases). Absence of the inguinal canal. Orchidopexy and primary repair	Favorable (6 m follow-up)
Rathore et al. (2012) [61] *****	USA	Five patients (9–15 y) (1) 15 y (2) 15 y (3) 13 y (4) 9 y (5) 11 y	All male	-	-	Three patients with associated visceral injury: patients (1) and (4): cecal wall hematoma; patient (2): duodenal hematoma, pancreatic contusion	Previous local trauma (bicycle handlebar) in all patients	-	(5) CT: Compatible with an SH	Surgical	-	(1) persistent pain. (2) pancreatic pseudocyst. Three cases evolved favorably
Decker et al. (2012) [62] *****	Germany	13 y	Male	Right	-	Abdominal wall hematoma	Previous local trauma (bicycle handlebar)	-	CT: 18.39 mm gap in the fascia of the abdominal rectus muscle and the internal and external oblique muscles with two intestinal loops	Surgical	SH. Primary repair	Favorable
Parihar et al. (2013) [63]	India	3 m	Male	Right	Right UDT	-	-	-	US: Testis in the layers of the abdominal wall, along with small bowel loops, echoes	Surgical	SH. Peritoneal sac present, containing the ipsilateral testis. Absence of the inguinal canal. Orchidopexy and primary repair	Favorable
Thakur et al. (2013) [64]	India	9 y	Male	Right	-	-	Previous local trauma (bicycle handlebar) 5 weeks before	6 × 4 cm (bulge), 4 × 4 cm (ring)	US: 4 × 4 cm defect along the right semilunar line with small bowel loops	Surgical	SH. Peritoneal sac present (opened). Primary repair (non-absorbable sutures)	Favorable (18 m follow-up)
Upasani et al. (2013) [65] *****	UK	12 y	Male	Left (upper)	-	-	Previous local trauma (BMX handlebar)	10 × 10 cm (bulge),2 cm (ring)	US: Abdominal wall hematoma. CT: 2 cm fascial defect with fat herniating through the defect	Conservative (partial resolution)	-	Favorable (6 m follow-up)
Balsara et al. (2014) [66]	USA	2 w	Male	Left	Left UDT	-	-	-	US: Normal-sized left testicle within the SH in the left lower quadrant with loops of bowel	Surgical	Exploratory laparoscopy: Vas deferens and spermatic vessels entering the hernia sac. The left testis was confirmed to lie within the sac itself. Open correction: SH. Hernial sac present, containing the left testis. Orchidopexy and primary repair	Favorable (7 m follow-up)
Spinelli et al. (2014) [67]	Italy	14 y	Female	Right	-	One-year history of recurrent abdominal pain	-	1.5 cm	US: Fascial defect in the right hemiabdomen.	Surgical	SH. Hernia lipoma. Peritoneal sac present, containing greater omentum. Reduction and primary repair	Favorable
Talutis et al. (2014) [68]	USA	(1) 9 y (2) 7 y (3) 11 y (4) 7 y	(1) Male (2) Male (3) Male (4) Female	(1) Left (2) Right (3) Left (4) Right	-	(1) Contusion to the mid-jejunum (2) Mesenteric defect in the ileocecal region (4) Ileal perforation	(1,3,4): Previous local trauma (bicycle handlebar) (2): ATV collision	(1) 4 × 3 cm	(1) CT: 4 × 3 cm fascial defect (SH) with a contusion to the mid-jejunum (4) CT: Handlebar sign. Evidence of a right lower quadrant abdominal wall defect with herniation	(1–3): Surgical	(1,3,4): Laparoscopic. Conversion to open surgery (2): Open surgery	(1–4): Favorable
Pederiva et al. (2015) [69] *****	Italy	9 y	Male	Left	-	Ileal perforation (not diagnosed at CT)	Previous local trauma (bicycle handlebar)	-	CT: Abdominal wall hematoma. Defect through the rectus sheath between the left *rectus abdominis* and the internal and external oblique. Intra-abdominal fat herniated through the defect	Surgical	Laparotomy. Intestinal resection. Primary repair (absorbable sutures)	Favorable
Montalvo et al. (2015) [70] *******	Spain	1 m	Male	Left	Left UDT	-	-	-	US (NR)	-	SH. Laparoscopic approach: Peritoneal sac present, containing the left testis. Orchidopexy. No primary repair	Favorable (unknown follow-up)
Volpe et al. (2016) [71] *****	Italy	(1) 8 y (2) 9 y	(1) Male (2) Male	(1) Right (2) Right	-	-	(1) Previous local trauma (bicycle handlebar) (2) Previous local trauma (bicycle handlebar)	(1) 1.5 cm (2) 1 cm	(1) US: 1.5 cm hernia between the rectus and the internal oblique with herniation of the omentum. (2) US: 1 cm defect with bowel herniation	(1) Conservative (2) Conservative	-	(1) Favorable (last control: 3 mm defect) (12 m follow-up) (2) Favorable (last control: 3 mm defect) (2 m follow-up)
Shea et al. (2017) [72]	USA	16 y	Male	Left	-	-	Previous local trauma (bicycle handlebar)	1 × 1 cm	CT: SH in the anterior left lower abdominal wall	Surgical	SH. No peritoneal sac. Primary repair (absorbable sutures)	Favorable
Kamal et al. (2017) [73]	India	2 y	Male	Right	Patchy frontal hair loss, left eye deviation, and bilateral UDT	-	-	-	US: Defect in the anterior abdominal wall lateral to the rectus muscle with herniation of preperitoneal fat. Absence of both testes in the scrotal sac. CT: SH with small bowel herniation. Both testes on the inguinal region	Surgical	SH. Peritoneal sac present. Primary repair. One month later, bilateral orchidopexy	Favorable
Rinaldi et al. (2017) [74] *****	Italy	(1) 12 y (2) 13 y	(1) Male (2) Male	(1) Left (2) Right	-	-	(1) Previous local trauma (bicycle fall) (2) Previous local trauma (bicycle fall)	(1) 5 cm (intraoperative)	(1) US: No peritoneal lesions; CT: Defect in the left anterior abdominal wall between the lateral margin of the left rectus and the medial margin of the ipsilateral oblique muscles (2) US: Right rectus hemorrhage, free liquid in the right lower quadrant. CT: Defect of the right anterior abdominal wall, with a slight separation between the right rectus and the oblique muscles	(1) Surgical (urgent) (2) Surgical (urgent)	(1) SH. Omentum identified under the skin. Primary repair (absorbable sutures). (2) Exploratory laparoscopy. Peritoneal repair (partial report)	(1) Favorable (2) Favorable
Sinopidis et al. (2018) [75]	Greece	Newborn	Male	Left	Bilateral inguinal hernias	-	-	-	US: Preperitoneal fat protruding through a defect of the transversalis fascia	Surgical	2 m: bilateral inguinal hernia repair. 5 m: SH. Preperitoneal fat adhered to the tip of the hernial sac. Primary repair	Favorable (18 m follow-up)
Sengar et al. (2018) [76]	India	(1) 12 y (2) 4 y (3) 4 y (4) 2.5 y (5) 2.3 y (6) 2 y (7) 1.6 y (8) 1 m (9) 1 m (10) 6 d	(1) Female (2) Female (3) Male (4) Male (5) Male (6) Male (7) Male (8) Female (9) Male (10) Male	(1) Bilateral (2) Right (3) Left (4) Bilateral (5) Right (6) Right (7) Right (8) Left (9) Right (10) Left	(5) Right UDT (6) Right UDT (7) Right UDT (9) Umbilical hernia, lumbar hernia (10) Hypospadias	-	-	-	-	Surgical	(3): SH containing the small bowel (5,6,7,10): SH containing testis. All testes were normal without epididymal-testicular dissociation. In all cases, orchidopexy was performed. Two cases could be brought down up to a high scrotal level only. All cases: Primary repair	Favorable (2 m–8 y follow-up)
Fai-So et al. (2018) [77] *****	Australia	10 y	Male	Left	-	Incarcerated sigmoid colon	Previous local trauma (bicycle handlebar)	-	CT: SH with a loop of sigmoid colon	Surgical (urgent)	Exploratory laparoscopy. Primary repair (laparoscopic). Absorbable sutures	Favorable (5 w follow-up)
Vega-Mata et al. (2019) [78]	Spain	13 y	Male	Right	-	Obesity (BMI 32.5)	-	-	US: 7 mm SH with fat herniation	Surgical	SH. Peritoneal sac containing omentum. Primary repair (laparoscopic). Non-absorbable sutures	Favorable (1 y follow-up)
Deshmukh et al. (2019) [79]	India	11 m	Male	Left	Left UDT	-	-	-	-	Surgical	SH. Laparoscopic repair: Peritoneal sac present, containing the left testis. Orchidopexy and primary repair	-
Nagara et al. (2020) [80]	Japan	Newborn	Male	Left	Right-sided inguinal hernia. Left UDT. ZC4H2-associated disorders: Congenital contractures of upper and lower extremities, hypokinesia, paraesophageal hiatal hernia	Small bowel incarceration	-	-	CT: Left SH with incarcerated small bowel. Right-sided inguinal hernia	Conservative	-	Death (SH incarceration, sepsis)
Taha et al. (2021) [81]	Saudi Arabia	50 d	Male	Right	Right UDT	Incarceration.	-	-	Plain abdominal X-ray: Distended bowel loops, gasless lower abdomen, and right lower quadrant lucency. US: Small amount of intraperitoneal free fluid and a loop of bowel herniated through the abdominal wall, defect; the defect was (10 mm) in diameter	Surgical (urgent)	SH. Peritoneal sac present containing fluid, small bowel loops, and the right testis. Reduction, orchidopexy, primary repair	Complication: scrotal infection (conservative management). Testicular atrophy (2 y follow-up)
García-Sanchez et al. (2021) [82] *****	Spain	11 y	Female	Right	-	-	Previous local trauma (bicycle handlebar)	2.4 × 1.8 cm	US: 2.4 × 1.8 cm defect between *rectus abdominis* and oblique/transversus with omentum and small bowel loops inside	Surgical	-	Favorable (1 w follow-up)
Sinacer et al. (2021) [83]	Algeria	Newborn	Male	Right	Right inguinal hernia (incarcerated), bilateral UDT, polydactyly (right hand), and anal stenosis (type 1 diabetic mother)	-	-	1.5 cm	-	Surgical	SH. Peritoneal sac present, containing small bowel loops and the left testis. Reduction, orchidopexy, primary repair (non-absorbable sutures)	-
Tahmri et al. (2021) [84]	Tunisia	9 y	Male	Right	-	-	Previous local trauma (bicycle handlebar)	2 cm	US: Right *rectus abdominis* hematoma. Diastasis between the lateral edge of the *rectus abdominis* and the ipsilateral oblique and transverse muscles, resulting in a 14 mm hernia sac containing omentum	Surgical	SH. Peritoneal sac present. Primary repair (absorbable sutures)	Favorable
Gonuguntla et al. (2022) [85]	India	3 m	Male	Left	Left UDT	-	-	-	US: Two abdominal wall defects: one in the left iliac fossa (1.6 cm) And other posterosuperior to the the first defect in the posterior abdominal wall, lateral to the kidney. (1.3 × 0.6 cm). Left testis not visible	Surgical	SH. Laparoscopic repair. Absence of the inguinal canal and gubernaculum. Orchidopexy and primary repair	Favorable (1 y follow-up)
Kropilak et al. (2022) [86]	USA	8 y	Male	Left	-	-	Previous local trauma (bicycle handlebar)	-	CT: Traumatic SH in the left lower quadrant with incarceration of loops of small bowel and associated stranding of surrounding tissues	Surgical (urgent)	Diagnostic laparoscopy. Laparotomy conversion. SH. Small bowel mesenteric injury with a devitalized jejunum. Intestinal resection and anastomosis. Primary repair (absorbable suture)	-
Okumuş et al. (2022) [87]	Turkey	Newborn	Male	Right	Right UDT	-	-	2–3 cm	Plain abdominal X-ray: Intestinal loops under the skin, US: Ventral hernia	Surgical	SH. Peritoneal sac present, containing the right testis. Orchidopexy, primary repair	Favorable (6 m follow-up)
Kangabam et al. (2023) [88]	India	17 y	Male	Right	-	-	Previous local trauma (motorcycle handlebar)	1 cm	US: 1 × 1 cm defect in the right Spigelian aponeurosis with herniating bowel loops. CT: Confirmation of findings	Conservative (patient’s choice)	-	-
Farina et al. (2024) [89]	Italy	4 m	Male	Left ******	Left UDT.	Large bowel incarceration	-		US: 5.8 mm hernia breach containing a sac with colon loop, adipose tissue, fluid collection, and a testicle	Surgical (urgent)	Primary repair and testicle repositioning in the scrotal sac	Favorable
Ablatt et al. (2025) [90]	USA	2 w	Male	Left	Left UDT, bilateral inguinal hernia, and umbilical hernia	-	-		US: Left inguinal hernia containing fluid and non-obstructive bowel. SH was not diagnosed	Surgical (urgent)	Open umbilical hernia repair, laparoscopic left orchidopexy, open SH repair, open left inguinal hernia repair, open right inguinal hernia repair	Favorable (6 w follow-up)

SH: Spigelian hernia; TAWH: Traumatic abdominal wall hernia; UDT: undescended testis; NR: not reported; y: years; m: months; d: days; w: weeks; cm: centimeters; ESR: elective surgical repair; CT: computed tomography; US: ultrasound; IC: intercostal; USA: United States of America; UK: United Kingdom; BMI: Body mass index. *: The original article could not be found, and the data reported correspond to other previous reviews; **: the histological study showed muscle atrophy with fatty infiltration; ***: the authors suspected that it was an incisional hernia (postoperative hernia); ****: siblings; *****: reported nonspecifically as a handlebar hernia or traumatic abdominal wall hernia (TAWH), but radiologically and clinically compatible with an SH; ******: the authors report inconsistent data in the manuscript, sometimes referring to left-sided laterality and other times to right-sided laterality. However, the overall interpretation of the case suggests that it involves left-sided laterality. The authors were contacted to resolve the matter; *******: The patient is classified as female in Table 1 of Montalvo et al., an implausibility considering the coexisting undescended testis (UDT). However, in the main text, the authors clarify that the patient is male, and we have therefore considered him as such in our analyses.

**Table 2:** Key sociodemographic and clinical findings from the review.

Variable	Key Findings
Total number of patients	123 patients
Sex distribution	106 males (86.2%), 17 females (13.8%)
Age at diagnosis	Median (interquartile range): 3 (0.25–9) years Range 0–21 years
Laterality	Right: 56 patients (45.5%) Left: 47 patients (38.2%) Bilateral: 13 patients (10.6%) Not reported: 7 patients (5.7%)
Association with undescended testis (UDT)	41 patients (33.3%)
Association with trauma	45 patients (36.6%) Most are due to bicycle handlebar injuries
Incarceration/strangulation rate	15 patients (12.2%)
Surgical treatment performed	95 patients (77.2%) -At least 15 (15.8%) were urgent
Laparoscopic approach	14 patients (14.7%) -8 with a complete laparoscopic repair (57.1%) -5 required conversion (35.7%) -1 was laparoscopically explored but not repaired (7.1%)
Conservative management attempted	8 patients (6.5%) -3 achieved complete resolution (37.5%) -3 achieved partial resolution (37.5%) -1 patient died (SH incarceration) (12.5%) -1 patient had no reported follow-up (12.5%)
Reported outcomes	Favorable in 95 patients (77.2%) (variable follow-up) -2 SH-related deaths reported (1.6%) -No outcomes reported in 15.4%

## Data Availability

The dataset used to carry out this study is attached as Supplementary File S2.

## Supplementary Materials

The following supporting information can be downloaded at: https://www.mdpi.com/article/10.3390/children12091120/s1. Document S1: Inclusion and exclusion criteria. Document S2: Dataset comprising all primary data extracted for the analyses.
