# Supplementary material for: Pediatric Spigelian Hernia and Spigelian–Cryptorchidism Syndrome: An Integrative Review
Source: Children (Basel). 2025 Aug 25;12(9):1120. doi: 10.3390/children12091120 (PMC12468178; doi:10.3390/children12091120)
Supplement: Supplementary file 1 [file children-12-01120-s001.zip › Supplementary File S1. Inclusion and exclusion criteria R1.pdf]

Supplementary File 1. Inclusion and Exclusion Criteria

Inclusion criteria

-Prospective or retrospective original studies, case series, or individual cases reporting patients with a final diagnosis of Spigelian hernia.

Exclusion criteria

-Duplicate or overlapping studies.

-Reviews, systematic reviews, consensus guidelines. In studies that combined a case report with a literature review, only the case report was considered for data extraction.

-Languages other than English or Spanish.

-Studies with no population of interest.

-Studies conducted in adult patients.
